# Successful Oral Health Interventions for Children Living in Vulnerable Circumstances – A Scoping Review

**DOI:** 10.1016/j.identj.2025.100855

**Published:** 2025-06-30

**Authors:** Awani Balasooriyan, Christine Dedding, Clarissa Calil Bonifácio, Ruben R. Lacroix, Kirsten A. van Gelderen – Ziesemer, Rik Gerritsen, Monique H. van der Veen

**Affiliations:** a Department of Preventive Dentistry, Academic Centre for Dentistry Amsterdam (ACTA), University of Amsterdam and VU Amsterdam, Amsterdam, the Netherlands; b Department of Ethics, Law and Humanities, Amsterdam University Medical Centre (UMC), Amsterdam, The Netherlands; c Department of Paediatric Dentistry, Academic Centre for Dentistry Amsterdam (ACTA), Amsterdam, University of Amsterdam and VU Amsterdam, The Netherlands; d Medical University Library, VU Amsterdam, Amsterdam, The Netherlands

**Keywords:** Children’s oral health, Oral health inequality, Child oral health interventions, Macro, Meso, Micro system level

## Abstract

Promoting equal opportunities for young children’s oral health is a global health priority. Several interventions have been developed to address persistent oral health inequalities. However, the understanding of how these interventions improve children’s oral health remains limited. This scoping review aims to explore researchers’ explanations for the success of interventions promoting oral health in young children (≤5 years) and their families living in vulnerable circumstances in Western countries to strengthen future interventions. A comprehensive search strategy was developed and applied to three electronic databases: PubMed, Embase.com, and Clarivate Analytics/Web of Science Core Collection. This yielded 21,415 articles, which were screened according to the Preferred Reporting Items for Systematic Reviews and Meta-Analyses Extension for Scoping Reviews guidelines. The included articles were analysed thematically using a macro, meso, and micro system level framework. From a total of 21,415 references, 251 were assessed for eligibility; 39 articles were included, covering 37 interventions. In particular, interventions implemented at the macro level (n = 1), macro-meso levels (n = 4) and macro-meso-micro levels (n = 10) seem to be the most promising. The researchers argue that interventions with a personalised, educational and culturally sensitive approach, delivered through interdisciplinary collaboration between professionals within and outside the dental sector, supported by adequate resources (e.g., appropriate time, funding, location), are key to success. Future interventions require a careful account of families' complex daily realities by intensifying collaboration between parents, community workers, school staff, dental professionals, primary care providers and government, providing training and balanced workloads for professionals and ensuring sufficient resources. According to the researchers, engaging with actors from macro, meso, and micro system levels is essential in child oral health promotion. Government leadership is needed to address oral health inequalities as a societal responsibility *in partnership with* families, their social network, food shops, (pre-)schools, dental practices, community and primary care organisations.

## Introduction

Poor oral health in childhood is a substantial global public health concern.^1^ Dental caries is the most prevalent oral disease affecting nearly fifty percent of young children worldwide.^2^, ^3^, ^4^ Caries may lead to a reduced quality of life for children, and a high economic burden for families and the wider society.^5^^,^^6^ Although caries is largely preventable, it reflects widespread socioeconomic and oral health inequalities.^1^^,^^7^ Children growing up in vulnerable circumstances are disproportionately affected by oral health problems.^8^^,^^9^ With vulnerable circumstances we refer to the context that places an individual in a vulnerable position, such as intersections of a limited social network, age, parental educational background, disability, poor health, low socioeconomic position (SEP), deprived living environment and migration background.^10^, ^11^, ^12^, ^13^ In fact, childhood caries is an important predictor of future caries incidence.^14^ Therefore, it is imperative to introduce healthy oral health practices at an early age.

Despite significant improvements in oral health in Western countries, inequalities in oral health among young children persist.^3^ In the Netherlands, caries prevalence is higher among 5-year-old children from underserved communities.^7^^,^^8^ Similar inequalities are found in the United Kingdom, where access to oral health care and healthy oral hygiene practices were less common among young children with an ethnic minority background.^15^^,^^16^ As recognised in the recent WHO Global Oral Health Action Plan, reducing oral health inequalities is a global health priority.^1^

Achieving equality in young children’s oral health requires effective interventions targeting children from an early age, with parents and/or caregivers playing a key role. Many interventions have been developed that address factors at different system levels, including the macro level (e.g., economic, political and social factors), the meso level (e.g., organisational and community factors) and/or the micro level (e.g., individual behavioural factors).^17^, ^18^, ^19^, ^20^, ^21^ Examples of macro level interventions include community water fluoridation or sugar-sweetened beverage tax.^18^^,^^21^ Meso level interventions entail supervised tooth brushing in day care and schools.^18^^,^^22^ Micro level interventions aim to improve individual behaviours, such as education to promote good oral hygiene.^17^^,^^19^ Although interventions at each system level have the potential to reduce inequalities among young children, particularly macro level interventions, their impact remains inconclusive.^21^^,^^23^, ^24^, ^25^, ^26^

Therefore, evaluating the impact of interventions that address oral health inequalities has become an important research priority. Several reviews have recently been conducted to provide insight into the impact of interventions on oral health inequalities in young children.^27^, ^28^, ^29^, ^30^, ^31^ However, these reviews provide limited knowledge on the reasons why interventions promote oral health in young children living in vulnerable circumstances. There is currently no comprehensive overview of the explanations proposed by researchers for the success of oral health interventions.^32^ It is important to build this knowledge base in order to better understand why interventions promote young children’s well-being and oral health. To address this knowledge gap, a research approach is needed to explore the extent and nature of the literature describing successful interventions targeting young children and their families living in vulnerable circumstances. A scoping review provides the appropriate methodology to identify the relevant interventions and explore researcher’s explanations for their success.^33^

This scoping review aims to explore researchers’ explanations for the success of interventions promoting oral health in young children (≤5 years) and their families living in vulnerable circumstances in Western countries in order to strengthen future intervention development. The results can be used for future research lines and public health policies aiming to achieve oral health equality among young children.

## Material and methods

This scoping review followed the Preferred Reporting Items for Systematic Reviews and Meta-Analyses extension for Scoping Reviews (Supplementary file A)^34^^,^^35^ and addressed the research question: *How do researchers explain the success of interventions promoting oral health in young children (≤5 years) and their families living in vulnerable circumstances in Western countries and what implications and responsibilities follow from this for future intervention development?*

### Search strategy

A comprehensive search was conducted in PubMed, Embase.com, and Clarivate Analytics/Web of Science Core Collection databases from inception to September 5, 2023, in collaboration with two medical information specialists (RRL and KAZ). The search strategy included controlled terms and free text terms for synonyms of ‘child' combined with adjacent terms of (‘vulnerable circumstances’ OR ‘population-based’) AND ((‘dental caries’ AND ‘intervention’) OR ‘dental prevention’). The complete search strategy is available in (Supplementary file B). The search was performed without restrictions on methodology, date, or language. Duplicate articles were excluded by two medical information specialists (RRL and KAZ) using Endnote X20.0.1 (Clarivate^tm^), following the Amsterdam Efficient Deduplication (AED)-method^36^ and the Bramer method.^37^

### Eligibility criteria

Articles were selected for data extraction and analysis based on the eligibility criteria as listed in Table 1.

**Table 1:** Eligibility criteria.

*Inclusion criteria* - Articles describing oral health interventions implemented in Western countries (North America, Europe and Oceania). - Articles describing oral health interventions targeting children (≤5 years) or their parents/caregivers who live in vulnerable circumstances. With vulnerable circumstances we refer to the context that places an individual in a vulnerable position, such as intersections of a limited social network, age, parental educational background, disability, poor health, low socioeconomic position (SEP), deprived living environment or migration background. - Articles reporting on interventions that have been successful in promoting young children’s oral health. - Articles in which researchers explain why interventions promote young children’s oral health. - Articles in which researchers provide suggestions on how interventions can be improved to address oral health inequalities among young children. *Exclusion criteria* - Articles describing data or published before 2000. - Articles describing oral public health interventions that do not focus on children aged 5 years or younger.Articles reporting on interventions without a focus on addressing oral health inequalities. - Articles describing interventions that are not implemented in Western countries. - Articles containing limited insight into researchers’ explanations to understand the positive impact of interventions and how interventions may be improved.

### Study selection

Two authors (AB and RG) independently screened titles and abstracts, and assessed full-text articles for eligibility.^38^ The selection of eligible articles required unanimous agreement between both authors. A third author (MV) was consulted to resolve any disagreements and reach a consensus.

### Data extraction

From the included articles, the following data were tabulated: author, year and country); age of children and description of vulnerable circumstances; name, system level and type of intervention; the positive direct and indirect impact of the intervention on children’s oral health; researchers’ explanations for the success of interventions and suggestions for future intervention development. Data were extracted independently by two authors (AB and RG). Any discrepancies were resolved by discussion or consultation with a third author (MV).

### Data analysis

The included articles were analysed using thematic analysis with Atlas.ti.^39^ A macro, meso, and micro system level framework was used to analyse the included interventions.^40^^,^^41^ First, each intervention was categorised based on its system level according to the definitions listed in Table 2.^18^^,^^19^^,^^21^^,^^22^^,^^42^^,^^43^ Second, researchers’ explanations were explored to understand why interventions were considered successful in improving young children’s oral health. Third, the researchers’ suggestions for future intervention development were identified. The thematic analysis was conducted independently by two authors (AB and RG) and discussed in consultation with the research team (MV, CB, CD).

**Table 2:** Macro, meso, micro system analytical framework.

The macro system level includes the regional, national or international environment. Macro level interventions target the broader structural determinants (e.g., social, economic or political factors) causing poor oral health. Examples are: - Policy change - Community water fluoridation - Sugar tax The meso system level refers to a community or organisation. Meso level interventions target factors in key settings for children to promote oral health such as child day care, schools or primary care organisations. Examples are: - Interdisciplinary collaboration between dentists and teachers - Supervised tooth brushing at school or child daycare - Fluoride varnish application provided by non-oral health professionals The micro system level includes the immediate surroundings of a child. Micro level interventions target individual behaviours of a child/parent (e.g., toothbrushing, diet and use of dental care) to improve oral health. Examples are: - Oral health education - Promotion of oral health protective behaviours

## Results

**Fig. 1:**
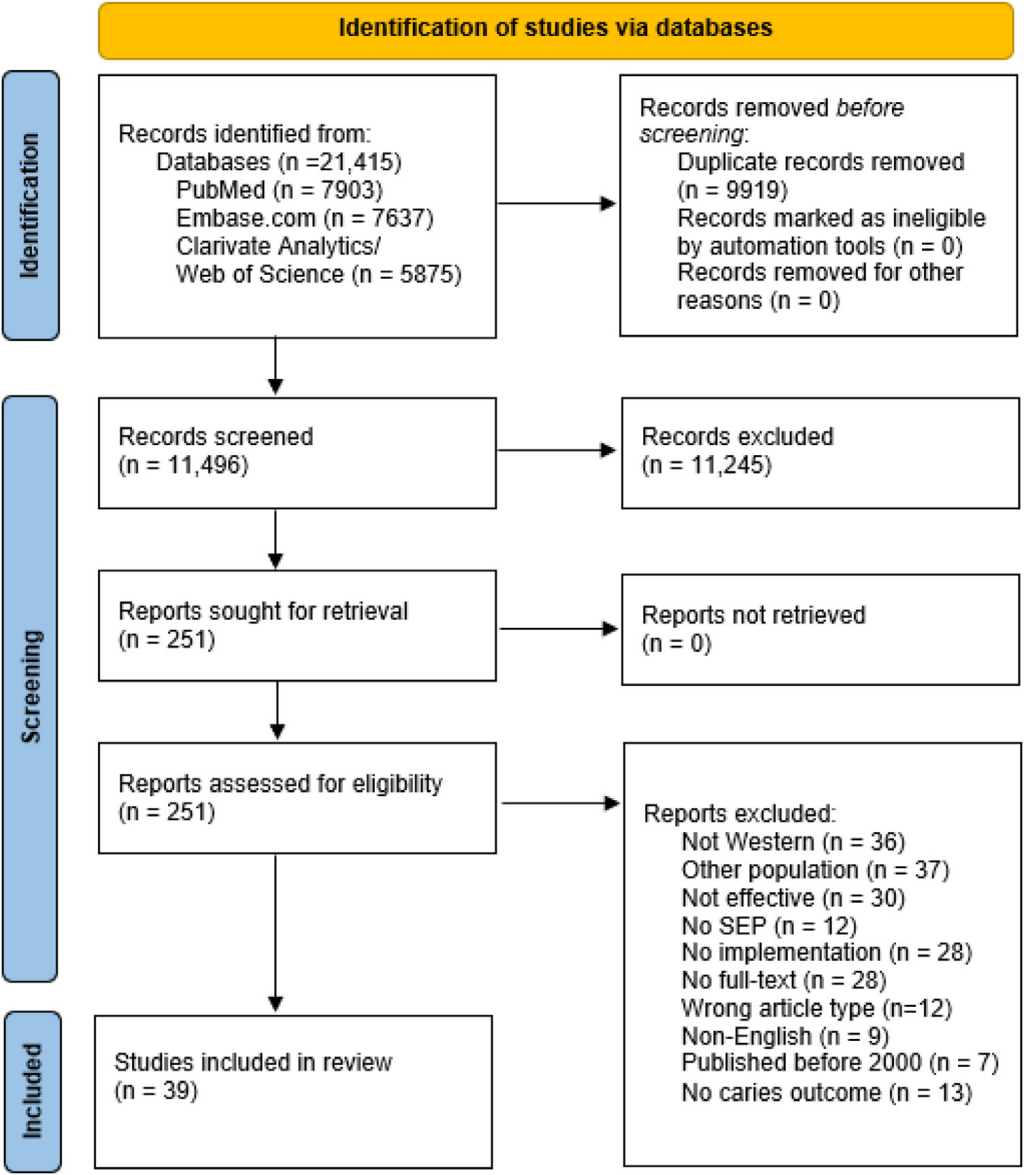
PRISMA flow diagram of article selection and screening.

The database searches yielded 21,415 articles. After the exclusion of duplicates, 11,496 titles and/or abstracts were quickly screened and the full texts of 251 articles were assessed. Finally, 39 articles met the inclusion criteria for successful interventions. Reasons for exclusion were interventions conducted in non-Western countries, interventions focused only on adults, articles describing data or published before 2000 and interventions that did not achieve the intended outcomes. The flow diagram for the screening and selection of relevant articles is shown in Figure 1.

### Overview of successful interventions

The 39 articles included covered 37 successful interventions implemented at one or multiple system levels. The articles were published between 2007 and 2023. A majority of the interventions were implemented in North America (n = 19) followed by Europe (n = 15) and Oceania (n = 2). Different age groups were targeted, ranging from pregnant women and newborns to children aged zero to six and three to seven years. One system level (n = 8) or multiple system levels (n = 31) were addressed by the interventions. The interventions were carried out in various settings, including local governments, primary care facilities, dental practices, (pre-) schools, kindergartens, nurseries, community centres and households. A diverse group of actors were involved in the implementation of the interventions, such as (regional) public health professionals, (oral) health care providers, teachers, community workers and parents/caregivers. The impact of the interventions varied from a direct impact on children’s oral health (e.g., lower caries levels, improved parental oral health behaviour) to an indirect impact that may contribute to improving children’s oral health (e.g., professional action, governmental action, intervention characteristics). A complete overview of the included articles and their (in)direct impact on children's oral health is provided in (Supplementary file C).

### Macro level interventions are most promising for children living in vulnerable circumstances

Government action is needed to improve young children’s oral health living in vulnerable circumstances. Therefore, interventions that address factors at the macro system level seem to be promising. Our results include one article describing a macro level intervention, namely community water fluoridation.^44^ This public health approach resulted in lower caries levels among 5-year-old children living in the most deprived areas of England, thereby contributing to the reduction of oral health inequalities.^44^ Water fluoridation is successful because it does not require individual behaviour change or compliance with health care advice.^44^ Interestingly, Roberts and colleagues^44^ provide little information on the role of the English government in their success.

Children living in vulnerable circumstances also benefit from interventions addressing macro-meso levels (n = 4) and macro-meso-micro levels (n = 10). With the exception of Okunseri and co-workers,^48^ all meso-micro interventions describe a positive impact on oral health inequalities.^45^, ^46^, ^47^ A government programme in North Carolina (USA) is beneficial because “*Programs targeting vulnerable populations through medical offices can reduce disparities in oral health among preschool aged children*.”^45^ Only Okunseri and co-workers^48^ and Brocklehurst and colleagues^46^ describe how government action contributed to their success. For example, the National Health Service in England played an important role in structuring the oral health programme entitled “*Baby Teeth DO Matter*” resulting in increased access to dental care.^46^

Two interventions implemented at macro-meso-micro levels describe an impact on oral health inequalities.^49^, ^50^, ^51^, ^52^ Wagner and co-researchers^52^ argue that the interdisciplinary approach of their oral health programme helps to reduce inequalities. The majority of the macro-meso-micro interventions (n = 6) helped to improve access to dental care.^49^, ^50^, ^51^, ^52^, ^53^, ^54^, ^55^, ^56^ The role of the government in the success of interventions is poorly described by Yuan and others^53^ and Evans and colleagues.^,^^57^ On the contrary, participation in national and regional programmes initiated by government institutions is promising for improving the oral health of children living in vulnerable circumstances.^49^, ^50^, ^51^, ^52^^,^^54^, ^55^, ^56^ The role of government institutions in providing funding and ensuring shared responsibilities is also critical to the success of interventions.^50^^,^^51^^,^^56^^,^^58^, ^59^, ^60^ An overview of all macro level interventions and their (in)direct impact is provided in Table 3.

**Table 3:** Macro level interventions and their (in)direct impact.

				Direct impact on child oral health								Indirect impact on child oral health								
				Children					Parents			Professionals				Govern-ment	Intervention			
Authors / Name intervention	Country	Target group -age children	System level(s) (key elements of intervention)	Improved oral health behaviours	Improved plaque scores	Increased access to dental care	Lower caries levels	Narrowing oral health inequalities	Improved oral health behaviours	Improved oral health in pregnancy	Increased oral health knowledge	Empowered professionals to promote oral health	Improved professional practice	Increased nursery-supervised tooth brushing	Increased rates of fluoride varnish applications	Statewide new regulations	High acceptance among child / parent	High participation among child / parent	Organisational satisfaction	Wide range of implementation opportunities
Roberts et al.^44^/ Community water fluoridation	UK	5 years	Macro level (fluoride in public water supplies)				**+**	**+**												
Achembong et al.^45^/ Into the Mouths of Babes	USA	0-4 years	Macro – Meso levels (a national programme of risk-based oral health counselling and fluoride varnish applications during medical office visits).				**+**	**+**							**+**			**+**		**+**
Brocklehorst et al.^46^/ Baby Teeth Do Matter	UK	< 5 years	Macro – Meso levels (a regional programme of active referral to dentists by local dental practices).			**+**		**+**				**+**								**+**
Okunseri et al.^48^/ Medicaid	USA	1-6 years	Macro – Meso levels (national programme to reimburse medical providers for fluoride varnish applications).												**+**					
Milsom et al.^47^/ Population prevention programme	UK	5 years	Macro – Meso levels (population programme of fluoride varnish applications and oral health materials provided by dentists).				**+**	**+**							**+**			**+**		
Yuan et al.^53^/ Treasure Baby Teeth	Ireland	0-5 years	Macro – Meso – Micro levels (government-led oral health education programme and active referral to dentists through home visits by health visitors).			**+**														
Giles et al.^60^/ HABIT	UK	9-12 months	Macro – Meso – Micro levels (government-led behavioural programme to promote infant toothbrushing through home visits by health visitors).		**+**					**+**							**+**	**+**		**+**
McMahon et al.^49^, Kidd et al.^50^, Ross et al. ^51^/ Childsmile	Scotland	Up to 5 years	Macro – Meso – Micro levels (a national programme of nursery-based fluoride varnish applications and supervised toothbrushing, dental health support worker home and community contacts, primary care dental practice visits).			**+**	**+**	**+**						**+**	**+**					
Biordi et al.^54^/ WIC program	USA	<5 years	Macro – Meso – Micro levels (national oral health education programme, fluoride varnish applications and active referral to dentists by nurses and dieticians).			**+**	**+**		**+**						**+**		**+**			
Burgette et al.^55^/ Early Head Start (EHS)	USA	< 3 years	Macro – Meso – Micro levels (national oral health education programme to promote fluoride and dental care use by EHS staff).			**+**												**+**		
Dudovitz et al. ^56^/ Head start programs	USA	2-4 years	Macro – Meso – Micro levels (national oral health literacy programme through training and reinforcement activities).	**+**		**+**			**+**		**+**	**+**						**+**		
Wagner et al. ^52^/ German Oral Health Programme	Germany	5 years	Macro – Meso – Micro levels (a regional programme of risk-based oral health counselling through home visits by health staff and fluoride varnish applications by dentists).			**+**	**+**	**+**	**+**						**+**			**+**		
Evans et al. ^57^/ Happy Teeth	UK	3-6 years	Macro – Meso – Micro levels (government-led oral health promotion programme and fluoride varnish applications at schools).												**+**		**+**	**+**	**+**	**+**
Huber et al.^58^/ Public fluoride varnish intervention	Canada	12-35 months; 6-8 years	Macro – Meso – Micro levels (a government-led programme of fluoride varnish applications in (pre-) schools by dentists).												**+**					
Hornsby et al.^59^/ Cavities Get around	USA	0-6 years	Macro – Meso – Micro levels (a government-led oral health education campaign to promote tap water using media and community workers).	**+**					**+**		**+**					**+**				

### Explanations for the success of interventions vary greatly

Researchers provide different explanations for the success of interventions that promote oral health in young children (≤5 years) living in vulnerable circumstances in Western countries. Some researchers go to great lengths to explain the success of their intervention, while others provide limited explanations. Most interventions include a combination of explanations focusing on either children, parents, professionals, government or intervention characteristics, as shown in (Supplementary file D). Key explanations include (1) A personalised, educational and culturally sensitive approach; (2) Interdisciplinary collaboration within and outside the dental sector; and (3) Adequate resources.

### Interventions should be personalised, educational and culturally sensitive

The majority of the researchers attribute their success to using a personalised, educational and culturally sensitive approach. A personalised approach is described as “*interventions delivered in this ‘family-friendly’ way […] may show promise to improve parental self-efficacy for child tooth brushing, behavioural intention for tooth brushing and dental attendance*.”^61^ Similarly, an intervention conveyed as “*person-to-person in repeated contacts*” resulted in improved parental behaviour.^62^ Gagnon and others^63^ add: “*The personalised nature of the intervention and the fact that a dental hygienist was available to answer any of the participants’ queries in a context of counselling facilitated the establishment of a bond of trust.*”

Many researchers indicate that an educational approach is highly valued. Parents from different ethnic backgrounds appreciate the educational tools (e.g., interactive PowerPoint presentation and a goal-setting card) used during their child’s dental appointment while they are waiting.^64^ Al-Jallad and co-researchers^65^ mention that their “*AICaries app*” is perceived as “*an useful educational tool for caries prevention*” and has improved parents’ oral health knowledge. According to Lee and colleagues,^66^ the use of oral health-related digital stories is an “*informative, educational, and a culturally safe tool for addressing ECC [Early Childhood Caries] and oral health care in young children*.”

Also, oral health interventions with a culturally sensitive approach are reported as beneficial for children living in vulnerable circumstances. The use of multilingual and multicultural materials helped to address family’s oral health needs.^67^ Hoeft and colleagues^68^ describe how having Spanish-speaking lay people leading the intervention allowed for high cultural sensitivity by drawing on community values. Hammersley and others^69^ highlight the importance of “*cultural safety*”, meaning that the intervention was safe and culturally appropriate for Indigenous families in Australia. Collaboration with Indigenous communities in Canada resulted in videos in which culture was a key factor in promoting oral health.^66^

Using a personalised, educational and culturally sensitive approach often helps to motivate parents to improve their children’s oral health. This includes parents actively working towards their self-set goal, parents being involved during intervention development, parents acting as research partners, and high parental attendance during an intervention. Huebner and colleagues^62^ praise their programme because “*It involved parents in its design and in its delivery. In the design phase, parents recommended active participation by parents*.” Well-received educational materials are considered to facilitate parental involvement.^64^ In addition, professional influences, reimbursement, and frequent contact moments increased parental participation leading to beneficial oral health outcomes.

However, some challenges have been identified in relation to a personalised, educational and culturally sensitive approach. Researchers struggle with language, literacy and communication barriers.^57^^,^^70^ The “*MySmileBuddy*” intervention addresses this issue by using a highly visual, bilingual iPad-based software and science-based, culturally specific education.^71^ Parental behaviour and the busy lives of families are also seen as stumbling blocks.^47^^,^^56^^,^^60^^,^^72^ A targeted approach tailored to the health needs of families living in vulnerable circumstances is recommended.^50^^,^^51^^,^^53^^,^^73^ “*Listening to parent’s narratives*” is therefore important.^74^ Giles and colleagues^60^ add: “ ‘*Liaising and working with parents closely will be key to the success of any future study*” complemented by giving “*monetary gifts*.”

### Successful interventions require interdisciplinary collaboration within and outside the dental sector

Another key explanation for the success of interventions relates to interdisciplinary collaboration, where professionals from different disciplines work together to improve the oral health of young children living in vulnerable circumstances. Researchers describe how collaboration between primary care providers, dental professionals, community workers, kindergarten employees, (pre-)school staff and government professionals, is essential. According to Dooley and colleagues,^75^ “*the development of an interdisciplinary team*” and their “*medical-dental-academic partnership*” contributed to their success.

The role of dental professionals in an interdisciplinary collaboration is most often reported. These range from dentists visiting kindergartens to help children brush their teeth, to dental teams working in a school.^76^^,^^77^ Also, an “*interprofessional partnership across nutrition and oral health disciplines*” helps to improve young children’s oral health.^78^ According to Purkis and co-researchers,^74^ collaboration between a social worker and a paediatric dentist is a mutual learning process that leads to good oral health outcomes. Collaboration with dental professionals is complicated by the shortage of dentists, insufficient communication skills and differences in dental working procedures and policies.^54^^,^^55^^,^^74^ Support is needed for dentists to help families living in vulnerable circumstances, such as working alongside a dental health support worker in the Childsmile intervention.^49^, ^50^, ^51^^,^^58^

Primary care providers are seen as important partners in promoting children’s oral health. They range from midwives, nurses, and physicians, to dieticians, social workers and prenatal care providers. The success of four interventions is entirely dependent on the influence of primary care providers.^56^^,^^72^^,^^79^^,^^80^ Group counselling by prenatal care providers resulted in beneficial oral health outcomes for pregnant women.^79^ In addition, Yuan and colleagues^53^ highlight the crucial role of health visitors as a “*healthcare bridge-builder*” between parents, children and dentists to increase access to dental care. Similarly, nurses and dieticians providing fluoride applications and dental referrals improved access to dentists.^54^ However, high workloads, public health cuts and reduced contact time with families can be problematic.^54^^,^^60^^,^^72^ Redistributing tasks, providing training in oral health, ensuring more time and sharing the workload between multiple professionals are recommended to address these issues.^54^^,^^60^^,^^75^^,^^76^

Other key players involved in the interdisciplinary collaboration are community professionals (i.e. community health workers, public health workers and community welfare workers) and school staff. Hammersley and colleagues^69^ highlight the important role of community professionals: “*The relationships established with Aboriginal Community Controlled Health Services and Aboriginal health workers were critical to successful community engagement.*” Working with a wide range of community partners in a statewide communication campaign has helped to improve the oral health of American families.^59^ School staff can play an essential role in allocating space and time for the intervention and providing valuable support.^58^ Yusuf and others^76^ found that a partnership between community professionals, school staff and dentists helped to better reach families. However, busy school environments, poor communication and a lack of a community-centred approach can be challenging.^65^^,^^76^ Therefore, collaboration with community health workers strengthened by sufficient time, space, manpower and clear communication will help.^65^^,^^76^

Government participation in interdisciplinary collaboration is mentioned in a few articles. The success of a community-based intervention depends on “*collaborative working between communities, health professionals and government agencies*” resulting in increased access to dental care.^53^ Huber and others^58^ describe the critical role of the Alberta Health Services public health services in increasing fluoride application rates through partnerships with dental professionals, schools and community workers. Collaboration with the UK National Health Service (NHS) is also important in child oral health promotion.^46^^,^^51^ Ross and co-researchers^51^ describe the success of the Childsmile intervention as “*This is led by the University of Glasgow in partnership with NHS Scotland and has had core funding from the Scottish Government*.” Adapting to new government structures can be a complicating factor.^46^ Huber and co-researchers^58^ state that sustained commitment of a government institution is needed by monitoring families’ needs and ongoing evaluations.

Explanations related to the important aspects of well-functioning interdisciplinary collaboration varied between the researchers. Staff training of the different professionals is seen as an essential component.^51^^,^^56^^,^^75^ Another key aspect is ensuring good communication between the professionals.^46^ A third factor is working with partners who can help with funding. Purkis and colleagues^74^ describe how their collaboration with a local foundation led to the financial feasibility of their programme. The need for more interdisciplinary collaboration in promoting children’s oral health is supported by the majority of researchers. Pieper and colleagues^77^ state that caries prevention requires “*interdisciplinary cooperation between obstetricians, midwives, paediatricians and dentists*.” This collaboration can be complemented by gynaecologists and social workers.^81^ The integration of oral health interventions into other community settings also seems promising.^76^

### Adequate resources are needed to support interventions

Researchers identify the availability of adequate resources to support an intervention as essential. This includes appropriate funding, time and location to successfully deliver an intervention. In particular, the availability of sufficient funding is critical. Brocklehurst and colleagues^46^ attribute the success of their programme to “*the necessary resources to support both clinicians and project management costs*.” Sufficient funding allows to hire an experienced project manager or to reimburse health professionals resulting in increased fluoride application rates.^45^^,^^48^^,^^51^^,^^75^ Providing reimbursement to families is also considered beneficial for improving young children’s oral health.^60^^,^^69^

Having enough time to carry out an intervention is also highly valued. According to Dooley and colleagues,^75^ the fact that their intervention fits into the flow of a primary care setting contributes to increased fluoride applications. A combined caries and obesity intervention is successful because it fits into the 60-minute appointments of an academic dental clinic setting.^78^ The use of the waiting room time is beneficial for parental oral health education.^64^ Al-Jallad and co-researchers^65^ identify the added value of artificial intelligence because “*it makes the lives of patients and healthcare providers easier by performing tasks in less time and less cost*.”

A suitable location to implement an intervention is another key requirement. Wenhall and others^82^ state that “*an easily accessible point of contact*” is critical to the delivery of their oral health outreach programme. School-based interventions ensure that children, who do not have regular access to dental care, have the opportunity to participate in dental prevention programmes.^57^^,^^76^ Evans and colleagues^57^ add “*If the school could facilitate, [fluoride] applications were undertaken in a suitable room rather than in a mobile dental clinic to reduce time and the cost of programme provision*.” In addition, interventions carried out in the home environment are highly beneficial.^60^^,^^65^^,^^69^^,^^80^

Concerns about funding mechanisms, time constraints and scarce resources are frequently mentioned. To overcome the barrier of limited financial resources, Purkis and others^74^ explain that “*We have partnered with a local non-profit foundation for 5 years now that provides partial financial support for the program*.” Time constraints can be addressed by “*flexibility of appointment scheduling and allocating adequate time*.”^69^ Gagnon and colleagues^63^ add: “*Removal of financial and physical barriers and personal professional involvement are good strategies to achieve compliance with fluoride supplements*.” Finally, rewarding families for their valuable time and providing them with the means to implement the newly learned oral health advice will help to improve young children’s oral health.^69^

## Discussion

This scoping review is the first to examine how researchers explain the reasons for the success of interventions promoting oral health in young children (≤5 years) and their families living in vulnerable circumstances in Western countries in order to strengthen future intervention development. The results of this study show that interventions implemented at the macro level (n = 1), macro-meso levels (n = 4) and macro-meso-micro levels (n = 10) seem to be the most promising. Researchers argue that interventions with a personalised, educational and culturally sensitive approach, delivered through interdisciplinary collaboration between professionals within and outside the dental sector, supported by adequate resources (e.g., appropriate time, funding, location), are key to success. Future interventions require a careful account of families' complex daily realities by intensifying collaboration between parents, community workers, school staff, dental professionals, primary care providers and government, providing training and balanced workloads for professionals, and ensuring sufficient resources.

First, while other studies have reported that government action and public health policy are key to addressing oral health inequalities,^32^^,^^83^ only fifteen interventions were identified targeting macro system level. From these, ten interventions resulted in a reduction in oral health inequalities or improved access to dental care. Surprisingly, researchers provide little information about the role of government and policy behind successful macro level interventions. According to Tsakos and colleagues,^32^ the direction and limited timeframe of funding programmes may not allow for research into the political factors that influence macro level interventions. A better understanding of government influence can help inform future interventions to develop national oral health policies tailored to the situational needs of children living in vulnerable circumstances.^84^ Systems thinking is an appropriate approach to gain insight into the complex and interdependent interactions between factors at the macro, meso, and micro system levels.^19^^,^^85^ This systems approach involves mixed methods and analytical tools (e.g., policy analysis and systems mapping) and can facilitate an understanding of government actions and policies that underpin the success of macro interventions.^32^

Second, although the governmental influence behind the success of interventions is not yet well understood, the researchers extensively describe the personalised, educational and culturally sensitive approach as a key success factor. It appears that most interventions have focused on individual responsibility, rather than societal responsibility.^86^ Particularly in the context of addressing oral health inequalities, individualised interventions need to be carefully considered.^87^ The vulnerable living circumstances of families are often influenced by environmental, social and political factors rather than individual behaviour.^88^ Individualised interventions focus mainly on oral health behaviours and education, but do not address the social and contextual factors that cause inequalities.^21^ Some studies have shown that individualised interventions may actually increase oral health inequalities.^23^^,^^24^ Children living in vulnerable circumstances are most likely to benefit from interventions that improve access to dental care or regulate food advertising.^19^ Therefore, there is an urgent need to move towards making young children's oral health a societal responsibility in which government influence is critical.^89^ Through government advocacy and funding, the development of interventions aimed at promoting equal opportunities in young children’s oral health can be achieved.^90^

Third, consistent with the literature,^91^^,^^92^ our study found that interdisciplinary collaboration between professionals within and outside the dental sector is key to the success of interventions. This can be seen as a promising first step towards making young children’s oral health a societal responsibility. In addition to the identified benefits of interdisciplinary collaboration between professionals, working together with families is critical to prioritise young children’s oral health. Ramji and colleagues^93^ highlight how a partnership between families, community professionals and representatives from the academic, public and private sectors, has influenced oral health behaviours. Our results show that community professionals in particular can play an important role in engaging families through their close contact with them. Similar findings are reported by Nghayo and others,^94^ demonstrating the value of community engagement programmes in improving oral health outcomes and expanding access to dental care. Therefore, important elements of future strategies to make young children’s oral health a societal responsibility include reflection and dialogue with families, professionals and government through a collaborative and ethically sound process of acting and working together.^93^^,^^95^

### Strengths and limitations

A key strength of our review is the comprehensive nature of the literature search. We aimed to identify all possible areas of oral health interventions. The involvement of several researchers in the process of reviewing and analysing the articles increased the scientific rigour of the findings. However, this review was not without its limitations. We only included English language peer-reviewed articles, book chapters or grey literature were excluded. To identify the explanations for the success of interventions, we were limited to what researchers reported in their articles. Implicit explanations that are not explicitly mentioned as success factors are not part of the findings, although they may have been relevant. For example, evaluation is an important aspect of the success of the Childsmile intervention.^51^ This relates to iterative learning but is not explicitly mentioned. In addition, the social and food environment has a major impact on young children's oral health,^13^^,^^96^ but we found little evidence of explanations addressing this.^65^ The inclusion of book chapters or grey literature might have enriched the findings by allowing researchers to describe and critically reflect on the success of their interventions in more detail.^97^ Another limitation is that this review focused only on successful interventions implemented in Western countries. Insights into the success of interventions developed in Asia, Africa or South America could be useful for strengthening future interventions. The data analysis approach was limited by the lack of consideration of contextual differences between countries. The large variability between the included articles posed a challenge for the analysis of country-specific health systems and public health policies. A better understanding of the contextual factors that cause oral health inequalities is key to developing interventions that are tailored to the needs of families living in vulnerable circumstances.

This scoping review has important implications for children’s oral health promotion. Our findings suggest that interventions based on a societal responsibility approach are needed to address oral health inequalities. This requires greater collaboration between families, their social environment, (local) food shops, community professionals, health professionals within and outside the dental sector and the government.^98^ In particular, government action is important to mobilise a range of actors to be part of this innovative partnership through funding and policy.^90^ In addition, targeted efforts are needed to increase the engagement of families in the development of interventions. It is important to listen to the situational needs of families living in vulnerable circumstances, build warm and trusting relationships and empower them.^99^

Our findings provide insights into the success of interventions and directions for future research. The explanations not specifically mentioned as success factors deserve further exploration, including explanations related to the food and social environment, the systems approach and the common risk factor approach, and the value of iterative learning during intervention development. We recommend adding these as search terms for a future scoping review. The search strategy can be extended with pregnancy-focused interventions to identify the value of introducing healthy oral health practices as early as possible.^14^ To build a comprehensive knowledge base, it will be beneficial to include ineffective oral health interventions. This will help to identify the facilitators and barriers of interventions aimed at promoting oral health in young children living in vulnerable circumstances.

In conclusion, government leadership is needed to address oral health inequalities in young children. This is a societal responsibility that should be undertaken in partnership with families, their social network, food shops, (pre-)schools, dental practices, community, and primary care organisations. By working together, a community approach can be co-created that may address poor oral health and overweight/obesity in young children simultaneously through a common risk factor approach.

## Conflict of interest

None disclosed.

## Appendix Supplementary materials

Image, application 1

Image, application 2

Image, application 3

Image, application 4

## Footnotes

Supplementary material associated with this article can be found in the online version at doi:10.1016/j.identj.2025.100855.
