# Supplementary material for: Successful Oral Health Interventions for Children Living in Vulnerable Circumstances – A Scoping Review
Source: Int Dent J. 2025 Jun 30;75(4):100855. doi: 10.1016/j.identj.2025.100855 (PMC12271906; doi:10.1016/j.identj.2025.100855)
Supplement: Supplementary file 2 [file mmc2.docx]

**Supplementary file B.** Complete search strategy

| **Search** | **PubMed Query – September 5, 2023** |
| --- | --- |
| #7 | #1 AND (#2 OR #3) AND ((#4 AND #5) OR #6) |
| #6 | "Preventive Dentistry"[Mesh] OR "Dental Care for Children"[Mesh] OR "fluoridat*"[tiab] OR "varnish*"[tiab] OR toothbrush*[tiab] OR teledentistry[tiab] OR "tele dentistry"[tiab] OR "preventive dentistry*"[tiab] OR "dental prevention*"[tiab] |
| #5 | "health education"[Mesh] OR "Health Promotion"[Mesh] OR health education*[tiab] OR intervention*[tiab] OR program*[tiab] OR "strateg*"[tiab] OR initiative*[tiab] OR support[tiab] OR advice[tiab] OR counseling[tiab] OR counselling[tiab] OR "assist*"[tiab] OR training[tiab] OR coaching[tiab] OR motivational-interview*[tiab] OR "instruct*"[tiab] OR "promot*"[tiab] OR "improv*"[tiab] OR "guidance*"[tiab] OR "educat*"[tiab] OR "teach*"[tiab] OR "influenc*"[tiab] OR "learn*"[tiab] OR "project*"[tiab] OR "action*"[tiab] OR "polic*"[tiab] OR develop*[tiab] OR implement*[tiab] OR stakeholder-engage*[tiab] OR public-healt[tiab] |
| #4 | "Oral Health"[Mesh] OR "Dental Caries"[Mesh] OR "oral health"[tiab] OR "Oral Hygiene"[tiab] OR "Oral safety"[tiab] OR "dental health"[tiab] OR "dental hygiene"[tiab] OR "dental safety"[tiab] OR "mouth hygiene"[tiab] OR "Caries"[tiab] OR "dental decay*"[tiab] OR "Carious Lesion*"[tiab] OR "dental white spot*"[tiab] OR "Carious Dentin*"[tiab] OR "plaque*"[tiab] |
| #3 | "School Health Services"[Mesh] OR "school health*"[tiab] OR "School-based"[tiab] OR "population-based"[tiab] OR "community-based"[tiab] OR "upstream"[tiab] OR downstream[tiab] OR midstream[tiab] OR public-health[tiab] |
| #2 | "Poverty"[Mesh] OR "Socioeconomic Factors"[Mesh] OR "Emigrants and Immigrants"[Mesh] OR "Refugees"[Mesh] OR "Transients and Migrants"[Mesh] OR "Minority Groups"[Mesh] OR ((socioeconomic*[tiab] OR "socio economic*"[tiab] OR economic*[tiab] OR "financial*"[tiab] OR "social*"[tiab] OR "vulnerab*"[tiab]) AND (class*[tiab] OR factor*[tiab] OR environment*[tiab] OR circumstance*[tiab] OR position*[tiab] OR barrier*[tiab])) OR socioeconomic*[tiab] OR "SES"[tiab] OR "SEP" [tiab] OR "living standard*"[tiab] OR "standard of living"[tiab] OR "income*"[tiab] OR financ*[tiab] OR "poverty"[tiab] OR "inequit*"[tiab] OR "inequalit*"[tiab] OR deprived[tiab] OR disparit*[tiab] OR Indigent [tiab] OR Indigency [tiab] OR literacy[tiab] OR "minorit*"[tiab] OR "migrant*"[tiab] OR "migration*"[tiab] OR immigrant*[tiab] OR refugee*[tiab] OR emigrant*[tiab] or "asylum seeker*"[tiab] OR "foreign national*"[tiab] OR ethnic*[tiab] |
| #1 | "Child, Preschool"[Mesh] OR "Infant"[Mesh] OR "neonat*"[tiab] OR "newborn*"[tiab] OR "new-born"[tiab] OR "infan*"[tiab] OR "baby*"[tiab] OR "babies"[tiab] OR "toddler*"[tiab] OR "child*"[tiab] OR "pediat*"[tiab] OR "paediat*"[tiab] OR "kid"[tiab] OR "kids"[tiab] OR "young*"[tiab] OR "youth*"[tiab] OR "girl*"[tiab] OR "boy"[tiab] OR "boys"[tiab] OR "nursery"[tiab] OR "daycare"[tiab] OR "day care"[tiab] OR "kindergarten*"[tiab] OR "early childhood education"[tiab] OR "preschool*"[tiab] OR "elementary education"[tiab] OR "primary education"[tiab] |
| **Search** | **Embase Query – September 5, 2023** |
| #8 | #7 NOT 'conference abstract'/it |
| #7 | #1 AND (#2 OR #3) AND (#4 AND #5 OR #6) |
| #6 | 'dental prevention'/exp OR 'mouth hygiene'/exp OR fluoridation*:ti,ab,kw OR varnish*:ti,ab,kw OR toothbrush*:ti,ab,kw OR teledentistry:ti,ab,kw OR 'tele dentistry':ti,ab,kw OR ((brush* NEAR/3 (tooth OR teeth)):ti,ab,kw) OR ((prevent* NEAR/3 dent*):ti,ab,kw) |
| #5 | 'health education'/exp OR educat*:ti,ab,kw OR intervention*:ti,ab,kw OR program*:ti,ab,kw OR strateg*:ti,ab,kw OR initiative*:ti,ab,kw OR support:ti,ab,kw OR advice:ti,ab,kw OR counseling:ti,ab,kw OR counselling:ti,ab,kw OR assist:ti,ab,kw OR training:ti,ab,kw OR coaching:ti,ab,kw OR 'motivational interview*':ti,ab,kw OR instruct*:ti,ab,kw OR promot*:ti,ab,kw OR improv*:ti,ab,kw OR guidance*:ti,ab,kw OR teach*:ti,ab,kw OR influenc*:ti,ab,kw OR learn*:ti,ab,kw OR project*:ti,ab,kw OR action*:ti,ab,kw OR polic*:ti,ab,kw OR develop*:ti,ab,kw OR implement*:ti,ab,kw OR 'stakeholder engage*':ti,ab,kw OR 'public health':ti,ab,kw |
| #4 | 'dental caries'/exp OR (((oral OR mouth OR dental) NEAR/3 (health* OR hygien* OR safety)):ti,ab,kw) OR caries:ti,ab,kw OR 'dental decay':ti,ab,kw OR carious:ti,ab,kw OR 'dental white spot':ti,ab,kw OR plaque:ti,ab,kw |
| #3 | 'school health service'/exp OR 'school health':ti,ab,kw OR 'school based':ti,ab,kw OR 'population based':ti,ab,kw OR 'community based':ti,ab,kw OR upstream:ti,ab,kw OR midstream:ti,ab,kw OR downstream:ti,ab,kw |
| #2 | 'socioeconomics'/exp OR 'migrant'/exp OR 'migration'/exp OR 'minority group'/exp OR (((socioeconomic* OR 'socio economic*' OR economic* OR financial* OR social* OR vulnerab*) NEAR/3 (class* OR factor* OR environment* OR circumstance* OR position* OR barrier*)):ti,ab,kw) OR socioeconomic*:ti,ab,kw OR ses:ti,ab,kw OR sep:ti,ab,kw OR 'living standard':ti,ab,kw OR 'standard of living':ti,ab,kw OR income*:ti,ab,kw OR finance*:ti,ab,kw OR poverty:ti,ab,kw OR inequit*:ti,ab,kw OR inequalit*:ti,ab,kw OR deprived:ti,ab,kw OR disparit*:ti,ab,kw OR indigent:ti,ab,kw OR indigenc*:ti,ab,kw OR literacy:ti,ab,kw OR minorit*:ti,ab,kw OR migrant*:ti,ab,kw OR migration*:ti,ab,kw OR immigrant*:ti,ab,kw OR refugee*:ti,ab,kw OR emigrant*:ti,ab,kw OR asylum:ti,ab,kw OR seeker*:ti,ab,kw OR 'foreign national*':ti,ab,kw OR ethnic:ti,ab,kw |
| #1 | 'preschool child'/exp OR 'toddler'/exp OR 'infant'/exp OR neonat*:ti,ab,kw OR newborn:ti,ab,kw OR 'new born':ti,ab,kw OR infan*:ti,ab,kw OR baby*:ti,ab,kw OR babies:ti,ab,kw OR toddler:ti,ab,kw OR child*:ti,ab,kw OR pediat*:ti,ab,kw OR paediat*:ti,ab,kw OR kid:ti,ab,kw OR kids:ti,ab,kw OR young:ti,ab,kw OR youth*:ti,ab,kw OR girl*:ti,ab,kw OR boy:ti,ab,kw OR boys:ti,ab,kw OR nursery:ti,ab,kw OR daycare:ti,ab,kw OR 'day care':ti,ab,kw OR kindergarten*:ti,ab,kw OR 'early childhood':ti,ab,kw OR 'pre school':ti,ab,kw OR preschool:ti,ab,kw OR 'elementary education':ti,ab,kw OR 'primary education':ti,ab,kw |

| **Search** | **Web of Science Query – September 5, 2023** |
| --- | --- |
| #7 | #1 AND (#2 OR #3) AND ((#4 AND #5) OR #6) |
| #6 | TS=("fluoridat*" OR "varnish*" OR toothbrush* OR (brush* NEAR/3 (tooth OR teeth)) OR teledentistry OR "tele dentistry" OR (prevent* NEAR/3 dent*)) |
| #5 | TS=(educati* OR intervention* OR program* OR "strateg*" OR initiative* OR support OR advice OR counseling OR counselling OR "assist*" OR training OR coaching OR motivational interview* OR "instruct*" OR "promot*" OR "improv*" OR "guidance*" OR "teach*" OR "influenc*" OR "learn*" OR "project*" OR "action*" OR "polic*" OR develop* OR implement* OR stakeholder-engage* OR public-health) |
| #4 | TS=("Caries" OR "dental decay*" OR "Carious*" OR "dental white spot*" OR "plaque*" OR ((oral* OR mouth OR dental) NEAR/3 (health* OR hygien* OR safety))) |
| #3 | TS=(“school health*” OR “School-based” OR “population-based” OR “community-based” OR upstream or downstream or midstream) |
| #2 | TS=((((socioeconomic* OR “socio economic*” OR economic* OR “financial*” OR "social*" OR vulnerab*) NEAR/3 (class* OR factor* OR environment* OR circumstance* OR position* OR barrier*)) OR socioeconomic* OR "SES" OR "SEP" OR "living standard*" OR "standard of living" OR "income*" OR financ* OR "poverty" OR "inequit*" OR "inequalit*" OR deprived OR disparit* OR Indigent OR Indigency OR literacy OR "minorit*" OR "migrant*" OR "migration*" OR immigrant* OR refugee* OR emigrant* or "asylum seeker*" OR "foreign national*" OR ethnic)) |
| #1 | TS=("neonat*" OR "newborn*" OR "new-born" OR "infan*" OR "baby*" OR "babies" OR "toddler*" OR "child*" OR "pediat*" OR "paediat*" OR "kid" OR "kids" OR "young*" OR "youth*" OR "girl*" OR "boy" OR "boys" OR "nursery" OR "daycare" OR "day care" OR "kindergarten*" OR "early childhood education" OR "preschool*" OR "elementary education" OR "primary education") |
