# Supplementary material for: Successful Oral Health Interventions for Children Living in Vulnerable Circumstances – A Scoping Review
Source: Int Dent J. 2025 Jun 30;75(4):100855. doi: 10.1016/j.identj.2025.100855 (PMC12271906; doi:10.1016/j.identj.2025.100855)
Supplement: Supplementary file 3 [file mmc3.docx]

| **Supplementary file C.** | | | | **Direct impact on child oral health** | | | | | | | | **Indirect impact on child oral health** | | | | | | | | |
| --- | --- | --- | --- | --- | --- | --- | --- | --- | --- | --- | --- | --- | --- | --- | --- | --- | --- | --- | --- | --- |
| Overview of (in)direct impact of interventions. | | | | **Children** | | | | | **Parents** | | | **Professionals** | | | | **Govern-ment** | **Intervention** | | | |
| **Authors /**  **Name**  **intervention** | **Country** | **Target group -age children** | **System level(s)**  **(key elements of intervention)** | **Improved oral health behaviours** | **Improved plaque scores** | **Increased access to dental care** | **Lower caries levels** | **Narrowing oral health inequalities** | **Improved oral health behaviours** | **Improved oral health in pregnancy** | **Increased oral health knowledge** | **Empowered professionals to promote oral health** | **Improved professional practice** | **Increased nursery-supervised tooth brushing** | **Increased rates of fluoride varnish applications** | **Statewide new regulations** | **High acceptance among child / parent** | **High participation among child / parent** | **Organisational satisfaction** | **Wide range of implementation opportunities** |
| Roberts et al. (2022)/ Community water fluoridation | UK | 5 years | Macro level  (fluoride in public water supplies) |  |  |  | **+** | **+** |  |  |  |  |  |  |  |  |  |  |  |  |
| Achembong et al. (2014)/  Into the Mouths of Babes | USA | 0-4 years | Macro – Meso levels  (national programme of risk-based oral health counselling and fluoride varnish applications during medical office visits). |  |  |  | **+** | **+** |  |  |  |  |  |  | **+** |  |  | **+** |  | **+** |
| Brocklehorst et al. (2013)/ Baby Teeth Do Matter | UK | < 5 years | Macro – Meso levels  (regional programme of active referral to dentist by local dental practices). |  |  | **+** |  | **+** |  |  |  | **+** |  |  |  |  |  |  |  | **+** |
| Okunseri et al. (2009)/ Medicaid | USA | 1-6 years | Macro – Meso levels  (national programme to reimburse medical providers for fluoride varnish applications). |  |  |  |  |  |  |  |  |  |  |  | **+** |  |  |  |  |  |
| Milsom et al. (2014)/ Population  prevention programme | UK | 5 years | Macro – Meso levels  (population programme of fluoride varnish applications and oral health materials provided by dentists). |  |  |  | **+** | **+** |  |  |  |  |  |  | **+** |  |  | **+** |  |  |

|  | | | | **Direct impact on child oral health** | | | | | | | | **Indirect impact on child oral health** | | | | | | | | |
| --- | --- | --- | --- | --- | --- | --- | --- | --- | --- | --- | --- | --- | --- | --- | --- | --- | --- | --- | --- | --- |
|  |  |  |  | **Children** | | | | | **Parents** | | | **Professionals** | | | | **Govern-ment** | **Intervention** | | | |
| **Authors /**  **Name**  **intervention** | **Country** | **Target group -age children** | **System level(s)**  **(key elements of intervention)** | **Improved oral health behaviours** | **Improved plaque scores** | **Increased access to dental care** | **Lower caries levels** | **Narrowing oral health inequalities** | **Improved oral health behaviours** | **Improved oral health in pregnancy** | **Increased oral health knowledge** | **Empowered professionals to promote oral health** | **Improved professional practice** | **Increased nursery-supervised tooth brushing** | **Increased rates of fluoride varnish applications** | **Statewide new regulations** | **High acceptance among child / parent** | **High participation among child / parent** | **Organisational satisfaction** | **Wide range of implementation opportunities** |
| Yuan et al. (2007)/ Treasure Baby Teeth | Ireland | 0-5 years | Macro – Meso – Micro levels  (government-led oral health education programme and active referral to dentist through home visits by health visitors). |  |  | **+** |  |  |  |  |  |  |  |  |  |  |  |  |  |  |
| Giles et al. (2022)/ HABIT | UK | 9-12 months | Macro – Meso – Micro levels  (government-led behavioural programme to promote infant toothbrushing through home visits by health visitors). |  | **+** |  |  |  |  | **+** |  |  |  |  |  |  | **+** | **+** |  | **+** |
| McMahon et al. (2011), Kidd et al. (2020), Ross et al. (2023)/ Childsmile | Scotland | Up to 5 years | Macro – Meso – Micro levels  (national programme of nursery-based fluoride varnish applications and supervised toothbrushing, dental health support worker home and community contacts, primary care dental practice visits). |  |  | **+** | **+** | **+** |  |  |  |  |  | **+** | **+** |  |  |  |  |  |
| Biordi et al. (2015)/ WIC program | USA | <5 years | Macro – Meso – Micro levels  (national oral health education programme, fluoride varnish applications and active referral to dentists by nurses and dieticians). |  |  | **+** | **+** |  | **+** |  |  |  |  |  | **+** |  | **+** |  |  |  |

|  | | | | **Direct impact on child oral health** | | | | | | | | **Indirect impact on child oral health** | | | | | | | | |
| --- | --- | --- | --- | --- | --- | --- | --- | --- | --- | --- | --- | --- | --- | --- | --- | --- | --- | --- | --- | --- |
|  |  |  |  | **Children** | | | | | **Parents** | | | **Professionals** | | | | **Govern-ment** | **Intervention** | | | |
| **Authors /**  **Name**  **intervention** | **Country** | **Target group -age children** | **System level(s)**  **(key elements of intervention)** | **Improved oral health behaviours** | **Improved plaque scores** | **Increased access to dental care** | **Lower caries levels** | **Narrowing oral health inequalities** | **Improved oral health behaviours** | **Improved oral health in pregnancy** | **Increased oral health knowledge** | **Empowered professionals to promote oral health** | **Improved professional practice** | **Increased nursery-supervised tooth brushing** | **Increased rates of fluoride varnish applications** | **Statewide new regulations** | **High acceptance among child / parent** | **High participation among child / parent** | **Organisational satisfaction** | **Wide range of implementation opportunities** |
| Burgette et al. (2017)/ Early Head Start (EHS) | USA | < 3 years | Macro – Meso – Micro levels  (national oral health education programme to promote fluoride and dental care use by EHS staff). |  |  | **+** |  |  |  |  |  |  |  |  |  |  |  | **+** |  |  |
| Dudovitz et al. (2020)/ Head start programs | USA | 2-4 years | Macro – Meso – Micro levels  (national oral health literacy programme through training and reinforcement activities). | **+** |  | **+** |  |  | **+** |  | **+** | **+** |  |  |  |  |  | **+** |  |  |
| Wagner et al. (2017)/ German Oral Health Programme | Germany | 5 years | Macro – Meso – Micro levels  (regional programme of risk-based oral health counselling through home visits by health staff and fluoride varnish applications by dentists). |  |  | **+** | **+** | **+** | **+** |  |  |  |  |  | **+** |  |  | **+** |  |  |
| Evans et al. (2013)/ Happy Teeth | UK | 3-6 years | Macro – Meso – Micro levels  (government-led oral health promotion programme and fluoride varnish applications at schools). |  |  |  |  |  |  |  |  |  |  |  | **+** |  | **+** | **+** | **+** | **+** |

|  | | | | **Direct impact on child oral health** | | | | | | | | **Indirect impact on child oral health** | | | | | | | | |
| --- | --- | --- | --- | --- | --- | --- | --- | --- | --- | --- | --- | --- | --- | --- | --- | --- | --- | --- | --- | --- |
|  |  |  |  | **Children** | | | | | **Parents** | | | **Professionals** | | | | **Govern-ment** | **Intervention** | | | |
| **Authors /**  **Name**  **intervention** | **Country** | **Target group -age children** | **System level(s)**  **(key elements of intervention)** | **Improved oral health behaviours** | **Improved plaque scores** | **Increased access to dental care** | **Lower caries levels** | **Narrowing oral health inequalities** | **Improved oral health behaviours** | **Improved oral health in pregnancy** | **Increased oral health knowledge** | **Empowered professionals to promote oral health** | **Improved professional practice** | **Increased nursery-supervised tooth brushing** | **Increased rates of fluoride varnish applications** | **Statewide new regulations** | **High acceptance among child / parent** | **High participation among child / parent** | **Organisational satisfaction** | **Wide range of implementation opportunities** |
| Huber et al. (2017)/ Public fluoride varnish intervention | Canada | 12-35 months; grade 1 and 2 | Macro – Meso – Micro levels  (government-led programme of fluoride varnish applications in (pre-) schools by dentists). |  |  |  |  |  |  |  |  |  |  |  | **+** |  |  |  |  |  |
| Hornsby et al. (2017)/ Cavities Get Around | USA | 0-6 years | Macro – Meso – Micro levels  (government-led oral health education campaign to promote tap water us by using media and community workers). | **+** |  |  |  |  | **+** |  | **+** |  |  |  |  | **+** |  |  |  |  |
| Amend et al. (2022)/ Early childhood caries preventive programme | Germany | 3-5 years | Meso level  (supervised tooth brushing, dental-check-ups and fluoride varnish applications in kindergartens by dentists). |  |  |  | **+** |  |  |  |  |  |  |  | **+** |  |  |  |  |  |
| Pieper et al. (2016)/ Intensified preventive programme | Germany | 2-4 years | Meso level  (supervised tooth brushing and dental check-ups in kindergartens by dentists). |  |  |  | **+** |  |  |  |  |  |  |  |  |  |  |  |  |  |

|  | | | | **Direct impact on child oral health** | | | | | | | | **Indirect impact on child oral health** | | | | | | | | |
| --- | --- | --- | --- | --- | --- | --- | --- | --- | --- | --- | --- | --- | --- | --- | --- | --- | --- | --- | --- | --- |
|  |  |  |  | **Children** | | | | | **Parents** | | | **Professionals** | | | | **Govern-ment** | **Intervention** | | | |
| **Authors /**  **Name**  **intervention** | **Country** | **Target group -age children** | **System level(s)**  **(key elements of intervention)** | **Improved oral health behaviours** | **Improved plaque scores** | **Increased access to dental care** | **Lower caries levels** | **Narrowing oral health inequalities** | **Improved oral health behaviours** | **Improved oral health in pregnancy** | **Increased oral health knowledge** | **Empowered professionals to promote oral health** | **Improved professional practice** | **Increased nursery-supervised tooth brushing** | **Increased rates of fluoride varnish applications** | **Statewide new regulations** | **High acceptance among child / parent** | **High participation among child / parent** | **Organisational satisfaction** | **Wide range of implementation opportunities** |
| Dooley et al. (2016)/ Oral health prevention | USA | 1-5 years | Meso level  (fluoride varnish applications, oral health education and active dental referral in primary care). |  |  |  |  |  |  |  |  |  |  |  | **+** |  |  |  |  | **+** |
| Yusuf et al. (2015)/ Keep Smiling | UK | 3-7 years | Meso level  (school-based fluoride varnish applications and brushing sessions by dentists and community workers). | **+** |  |  |  |  | **+** |  |  | **+** |  |  | **+** |  | **+** | **+** | **+** | **+** |
| Chomitz et al. (2019)/ Baby steps to health | USA | 6-36 months | Meso – Micro levels  (obesity and caries prevention programme to improve dietary behaviour via motivational interviewing in dental clinic). |  |  |  |  |  | **+** |  |  |  |  |  |  |  | **+** | **+** | **+** |  |
| Maupomé et al. (2010)/ Toddler Overweight & Tooth Decay Prevention Study | USA | 18-30 months | Meso – Micro levels  (community and family intervention aimed at reducing sugar-sweetened drinks and promoting breastfeeding and water). |  |  |  | **+** |  |  |  |  |  |  |  |  |  | **+** |  |  |  |

|  | | | | **Direct impact on child oral health** | | | | | | | | **Indirect impact on child oral health** | | | | | | | | |
| --- | --- | --- | --- | --- | --- | --- | --- | --- | --- | --- | --- | --- | --- | --- | --- | --- | --- | --- | --- | --- |
|  |  |  |  | **Children** | | | | | **Parents** | | | **Professionals** | | | | **Govern-ment** | **Intervention** | | | |
| **Authors /**  **Name**  **intervention** | **Country** | **Target group -age children** | **System level(s)**  **(key elements of intervention)** | **Improved oral health behaviours** | **Improved plaque scores** | **Increased access to dental care** | **Lower caries levels** | **Narrowing oral health inequalities** | **Improved oral health behaviours** | **Improved oral health in pregnancy** | **Increased oral health knowledge** | **Empowered professionals to promote oral health** | **Improved professional practice** | **Increased nursery-supervised tooth brushing** | **Increased rates of fluoride varnish applications** | **Statewide new regulations** | **High acceptance among child / parent** | **High participation among child / parent** | **Organisational satisfaction** | **Wide range of implementation opportunities** |
| Wenhall et al. (2008)/ Oral health outreach programme | Sweden | 2-5 years | Meso – Micro levels  (community-based oral health education and free fluoride tablets by dentists in an outreach facility). |  |  |  | **+** |  | **+** |  |  |  |  |  |  |  | **+** | **+** |  |  |
| Neumann et al. (2011)/ Country KIDS | Australia | 7-8 months until 3 years | Meso – Micro levels  (nurses provide oral health materials and education through home visits). |  |  |  | **+** |  |  |  |  |  |  |  |  |  |  |  |  |  |
| Adams et al. (2017)/ Centering  Pregnancy | USA | Preg-nant women | Meso – Micro levels  (oral health education using a skills-building approach during group prenatal care sessions). |  |  |  |  |  |  | **+** | **+** |  |  |  |  |  |  |  |  |  |
| Soussou et al. (2017)/ Dental education program | Canada | < 6 years | Meso – Micro levels  (‘waiting-room based’ oral health education to engage parents during their child's dental appointment). |  |  |  |  |  | **+** |  |  |  |  |  |  |  | **+** |  |  |  |

|  | | | | **Direct impact on child oral health** | | | | | | | | **Indirect impact on child oral health** | | | | | | | | |
| --- | --- | --- | --- | --- | --- | --- | --- | --- | --- | --- | --- | --- | --- | --- | --- | --- | --- | --- | --- | --- |
|  |  |  |  | **Children** | | | | | **Parents** | | | **Professionals** | | | | **Govern-ment** | **Intervention** | | | |
| **Authors /**  **Name**  **intervention** | **Country** | **Target group -age children** | **System level(s)**  **(key elements of intervention)** | **Improved oral health behaviours** | **Improved plaque scores** | **Increased access to dental care** | **Lower caries levels** | **Narrowing oral health inequalities** | **Improved oral health behaviours** | **Improved oral health in pregnancy** | **Increased oral health knowledge** | **Empowered professionals to promote oral health** | **Improved professional practice** | **Increased nursery-supervised tooth brushing** | **Increased rates of fluoride varnish applications** | **Statewide new regulations** | **High acceptance among child / parent** | **High participation among child / parent** | **Organisational satisfaction** | **Wide range of implementation opportunities** |
| Hoeft et al. (2016)/ Contra Caries | USA | 0-5 years | Meso – Micro levels  (oral health education provided by community workers targeting Spanish-speaking parents). |  |  |  |  |  | **+** |  | **+** |  |  |  |  |  | **+** | **+** |  |  |
| Huebner et al. (2014)/ Taking Care of Baby Teeth | USA | 0-6 years | Meso – Micro levels  (a parent-designed programme to support tooth brushing by community workers and dentists). |  |  |  |  |  | **+** |  | **+** |  |  |  |  |  |  |  |  |  |
| Brännemo et al. (2020)/ An extended postnatal programme | Sweden | 1/2 weeks-36 months | Meso – Micro levels  (a parental support home visiting programme on oral health by nurses, parental advisors and dentists. |  |  |  | **+** |  | **+** |  |  |  |  |  |  |  |  |  |  |  |
| Wagner et al. (2014)/ Oral health promotion programme | Austria | 5 years | Meso – Micro levels  (dental counselling for new mothers by dental health educators in regional hospitals). |  |  | **+** | **+** |  | **+** |  | **+** |  |  |  |  |  |  |  |  |  |

|  | | | | **Direct impact on child oral health** | | | | | | | | **Indirect impact on child oral health** | | | | | | | | |
| --- | --- | --- | --- | --- | --- | --- | --- | --- | --- | --- | --- | --- | --- | --- | --- | --- | --- | --- | --- | --- |
|  |  |  |  | **Children** | | | | | **Parents** | | | **Professionals** | | | | **Govern-ment** | **Intervention** | | | |
| **Authors /**  **Name**  **intervention** | **Country** | **Target group -age children** | **System level(s)**  **(key elements of intervention)** | **Improved oral health behaviours** | **Improved plaque scores** | **Increased access to dental care** | **Lower caries levels** | **Narrowing oral health inequalities** | **Improved oral health behaviours** | **Improved oral health in pregnancy** | **Increased oral health knowledge** | **Empowered professionals to promote oral health** | **Improved professional practice** | **Increased nursery-supervised tooth brushing** | **Increased rates of fluoride varnish applications** | **Statewide new regulations** | **High acceptance among child / parent** | **High participation among child / parent** | **Organisational satisfaction** | **Wide range of implementation opportunities** |
| Hammersley et al. (2022)/ Baby teeth talk | Australia | Preg-nancy until 5 years | Meso – Micro levels  (pregnancy dental care and counselling, fluoride varnish applications and motivational interviewing in the community). |  |  |  | **+** |  | **+** |  | **+** |  |  |  | **+** |  |  |  |  |  |
| Gagnon et al. (2007)/ Compliance with fluoride supplements | Canada | 6-9 months | Meso – Micro levels  (provision of fluoride supplements at family's homes by dental hygienists). |  |  |  |  |  | **+** |  |  |  |  |  |  |  | **+** | **+** |  |  |
| Purkis et al. (2023)/ Pediatric dental residency program | USA | 0-3 years | Meso – Micro levels  (social work programme of active dental referral and interprofessional practice in a pediatric dental clinic). |  |  | **+** |  |  |  |  |  |  | **+** |  |  |  |  |  | **+** |  |
| Cunha-Cruz et al. (2017)/ Everybody Brush! | USA | < 36 months (1-3 years) | Meso – Micro levels  (home delivery of oral health education and materials provided by dental organisation). |  |  |  |  |  |  |  |  |  |  |  |  |  | **+** |  | **+** |  |

|  | | | | **Direct impact on child oral health** | | | | | | | | **Indirect impact on child oral health** | | | | | | | | |
| --- | --- | --- | --- | --- | --- | --- | --- | --- | --- | --- | --- | --- | --- | --- | --- | --- | --- | --- | --- | --- |
|  |  |  |  | **Children** | | | | | **Parents** | | | **Professionals** | | | | **Govern-ment** | **Intervention** | | | |
| **Authors /**  **Name**  **intervention** | **Country** | **Target group -age children** | **System level(s)**  **(key elements of intervention)** | **Improved oral health behaviours** | **Improved plaque scores** | **Increased access to dental care** | **Lower caries levels** | **Narrowing oral health inequalities** | **Improved oral health behaviours** | **Improved oral health in pregnancy** | **Increased oral health knowledge** | **Empowered professionals to promote oral health** | **Improved professional practice** | **Increased nursery-supervised tooth brushing** | **Increased rates of fluoride varnish applications** | **Statewide new regulations** | **High acceptance among child / parent** | **High participation among child / parent** | **Organisational satisfaction** | **Wide range of implementation opportunities** |
| Lumsden (2019) / MySmile-Buddy | USA | 2-6 years | Meso – Micro levels  (i-Pad-based oral health education and behaviour change approach delivered by dentists). |  |  |  |  |  | **+** |  |  |  |  |  |  |  |  | + |  |  |
| O'Malley et al. (2017)/  Kitten’s First Tooth | UK | 4 years | Micro level  (a children’s storybook to improve parental attitudes self-efficacy towards their brushing and diet). |  |  |  |  |  | **+** |  |  |  |  |  |  |  | **+** |  |  |  |
| Lee et al (2023)/ My first teeth | Canada | < 6 years | Micro level  (oral health-related digital story videos in which parents share their experiences to deal with caries at home). |  |  |  |  |  | **+** |  |  |  |  |  |  |  |  |  |  | **+** |
| Al-Jallad (2022) / AICaries | USA | 1-5 years | Micro level  (artificial intelligence-based smartphone app caries detection, and personalised oral health education). |  |  |  |  |  |  |  | **+** |  |  |  |  |  | **+** |  |  |  |
