# Supplementary material for: Successful Oral Health Interventions for Children Living in Vulnerable Circumstances – A Scoping Review
Source: Int Dent J. 2025 Jun 30;75(4):100855. doi: 10.1016/j.identj.2025.100855 (PMC12271906; doi:10.1016/j.identj.2025.100855)
Supplement: Supplementary file 4 [file mmc4.docx]

| **Supplementary file D.** Overview of explanations related to the success of an interventions. | | | | | | | | | | | | | | | | | | | | | | | | | | | | | |
| --- | --- | --- | --- | --- | --- | --- | --- | --- | --- | --- | --- | --- | --- | --- | --- | --- | --- | --- | --- | --- | --- | --- | --- | --- | --- | --- | --- | --- | --- |
|  |  | **Children** | | **Parents** | | | | | **Professionals** | | | **Government** | | | | **Intervention** | | | | | | | | | | | | | |
| **Authors /**  **Name**  **intervention** | **System levels(s)** | **Willingness to cooperate** | **Active involvement** | **Sharing experiences & helping others** | **Engagement and self-efficacy** | **High interest in oral health** | **Motivation to change behaviour** | **Active involvement** | **Training** | **Interdisciplinary collaboration** | **Active involvement** | **Governmental input** | **Nationwide program** | **Interdisciplinary collaboration** | **Active involvement** | **Adaptability** | **Available resources** | **Observed advantage** | **Design and content** | **Accessibility and usability** | **Feasibility** | **Population approach** | **Community approach** | **Social support / group approach** | **Personalised / family approach** | **Building rapport & trust with families** | **Educational approach for families** | **Distribution of oral health materials** | **Media use** |
| Roberts et al. (2022)/ Community water fluoridation | Macro level |  |  |  |  |  |  |  |  |  |  | **+** |  |  |  |  |  |  | **+** |  |  |  |  |  |  |  |  |  |  |
| Achembong et al. (2014)/  Into the Mouths of Babes | Macro – Meso levels |  |  |  |  |  |  |  |  |  | **+** |  | **+** |  |  |  | **+** | **+** |  |  |  |  |  |  |  |  |  |  |  |
| Brocklehorst et al. (2013)/ Baby Teeth Do Matter | Macro – Meso levels |  |  |  |  |  |  |  |  | **+** | **+** | **+** |  | **+** |  |  | **+** |  |  | **+** |  |  |  |  |  |  |  | **+** |  |
| Okunseri et al. (2009)/ Medicaid | Macro – Meso levels |  |  |  |  |  |  |  |  |  | **+** | **+** |  |  |  |  |  |  |  |  |  |  |  |  |  |  |  |  |  |
| Milsom et al. (2014)/ Population  prevention programme | Macro – Meso levels |  |  |  |  |  |  |  |  |  | **+** | **+** |  |  |  |  | **+** | **+** |  |  |  |  |  |  | **+** |  |  |  |  |

|  | | | | | | | | | | | | | | | | | | | | | | | | | | | | | |
| --- | --- | --- | --- | --- | --- | --- | --- | --- | --- | --- | --- | --- | --- | --- | --- | --- | --- | --- | --- | --- | --- | --- | --- | --- | --- | --- | --- | --- | --- |
|  |  | **Children** | | **Parents** | | | | | **Professionals** | | | **Government** | | | | **Intervention** | | | | | | | | | | | | | |
| **Authors /**  **Name**  **intervention** | **System levels(s)** | **Willingness to cooperate** | **Active involvement** | **Sharing experiences & helping others** | **Engagement and self-efficacy** | **High interest in oral health** | **Motivation to change behaviour** | **Active involvement** | **Training** | **Interdisciplinary collaboration** | **Active involvement** | **Governmental input** | **Nationwide program** | **Interdisciplinary collaboration** | **Active involvement** | **Adaptability** | **Available resources** | **Observed advantage** | **Design and content** | **Accessibility and usability** | **Feasibility** | **Population approach** | **Community approach** | **Social support / group approach** | **Personalised / family approach** | **Building rapport & trust with families** | **Educational approach for families** | **Distribution of oral health materials** | **Media use** |
| Yuan et al. (2007)/ Treasure Baby Teeth | Macro – Meso – Micro levels | \| **-** \|  \|  \|  \|  \|  \|  \|  \|  \|  \|  \|  \|  \|  \|  \|  \|  \| \| --- \| --- \| --- \| --- \| --- \| --- \| --- \| --- \| --- \| --- \| --- \| --- \| --- \| --- \| --- \| --- \| --- \| | **+** |  |  |  |  | **+** | **+** | **+** | **+** | **+** |  | **+** |  |  |  |  |  |  |  |  |  |  | **+** |  | **+** | **+** |  |
| Giles et al. (2022)/ HABIT | Macro – Meso – Micro levels |  |  |  |  |  |  | **+** | **+** |  | **+** |  |  |  | **+** |  | **+** |  | **+** | **+** | **+** |  |  |  | **+** |  | **+** | **+** |  |
| McMahon et al. (2011), Kidd et al. (2020), Ross et al. (2023)/ Childsmile | Macro – Meso – Micro levels |  | **+** |  |  |  | **+** | **+** | **+** | **+** | **+** | **+** | **+** | **+** | **+** | **+** | **+** | **+** | **+** |  |  | **+** |  |  | **+** |  |  |  |  |
| Biordi et al. (2015)/ WIC program | Macro – Meso – Micro levels |  |  |  |  | **+** |  |  | **+** | **+** | **+** |  | **+** | **+** |  |  | **+** |  |  |  |  |  |  |  |  |  | **+** | **+** |  |
| Burgette et al. (2017)/ Early Head Start (EHS) | Macro – Meso – Micro levels |  |  |  |  |  |  |  | **+** |  | **+** |  | **+** |  |  |  |  |  | **+** |  |  |  |  |  | **+** |  |  |  |  |

|  | | | | | | | | | | | | | | | | | | | | | | | | | | | | | |
| --- | --- | --- | --- | --- | --- | --- | --- | --- | --- | --- | --- | --- | --- | --- | --- | --- | --- | --- | --- | --- | --- | --- | --- | --- | --- | --- | --- | --- | --- |
|  |  | **Children** | | **Parents** | | | | | **Professionals** | | | **Government** | | | | **Intervention** | | | | | | | | | | | | | |
| **Authors /**  **Name**  **intervention** | **System levels(s)** | **Willingness to cooperate** | **Active involvement** | **Sharing experiences & helping others** | **Engagement and self-efficacy** | **High interest in oral health** | **Motivation to change behaviour** | **Active involvement** | **Training** | **Interdisciplinary collaboration** | **Active involvement** | **Governmental input** | **Nationwide program** | **Interdisciplinary collaboration** | **Active involvement** | **Adaptability** | **Available resources** | **Observed advantage** | **Design and content** | **Accessibility and usability** | **Feasibility** | **Population approach** | **Community approach** | **Social support / group approach** | **Personalised / family approach** | **Building rapport & trust with families** | **Educational approach for families** | **Distribution of oral health materials** | **Media use** |
| Dudovitz et al. (2020)/ Head start programs | Macro – Meso – Micro levels | **+** |  |  | **+** |  |  | **+** | **+** | **+** | **+** |  | **+** | **+** | **+** | **+** | **+** | **+** | **+** |  |  | **+** |  |  | **+** | **+** | **+** | **+** | **+** |
| Wagner et al. (2017)/ German Oral Health Programme | Macro – Meso – Micro levels |  |  |  |  |  | **+** | **+** | **+** | **+** | **+** |  | **+** |  |  |  |  | **+** | **+** |  |  |  |  |  |  |  | **+** | **+** |  |
| Evans et al. (2013)/ Happy Teeth | Macro – Meso – Micro levels |  |  |  |  |  |  | **+** | **+** | **+** | **+** | **+** |  |  |  | **+** | **+** |  | **+** | **+** |  |  |  |  | **+** |  |  | **+** |  |
| Huber et al. (2017)/ Public fluoride varnish intervention | Macro – Meso – Micro levels |  |  |  |  |  |  | **+** |  | **+** | **+** |  |  | **+** | **+** |  |  |  |  |  |  | **+** |  | **+** |  |  |  |  | **+** |
| Hornsby et al. (2017)/ Cavities Get Around | Macro – Meso – Micro levels |  |  |  |  |  | **+** | **+** | **+** | **+** | **+** |  |  | **+** | **+** | **+** |  | **+** | **+** |  |  |  | **+** |  |  |  | **+** | **+** | **+** |

|  | | | | | | | | | | | | | | | | | | | | | | | | | | | | | |
| --- | --- | --- | --- | --- | --- | --- | --- | --- | --- | --- | --- | --- | --- | --- | --- | --- | --- | --- | --- | --- | --- | --- | --- | --- | --- | --- | --- | --- | --- |
|  |  | **Children** | | **Parents** | | | | | **Professionals** | | | **Government** | | | | **Intervention** | | | | | | | | | | | | | |
| **Authors /**  **Name**  **intervention** | **System levels(s)** | **Willingness to cooperate** | **Active involvement** | **Sharing experiences & helping others** | **Engagement and self-efficacy** | **High interest in oral health** | **Motivation to change behaviour** | **Active involvement** | **Training** | **Interdisciplinary collaboration** | **Active involvement** | **Governmental input** | **Nationwide program** | **Interdisciplinary collaboration** | **Active involvement** | **Adaptability** | **Available resources** | **Observed advantage** | **Design and content** | **Accessibility and usability** | **Feasibility** | **Population approach** | **Community approach** | **Social support / group approach** | **Personalised / family approach** | **Building rapport & trust with families** | **Educational approach for families** | **Distribution of oral health materials** | **Media use** |
| Amend et al. (2022)/ Early childhood caries preventive programme | Meso level |  |  |  |  |  |  |  |  | **+** | **+** |  |  |  |  |  |  |  |  |  | **+** |  |  |  |  |  |  |  |  |
| Pieper et al. (2016)/ Intensified preventive programme | Meso level |  |  |  |  |  |  |  |  | **+** | **+** |  |  |  |  |  |  |  |  |  |  |  |  |  | **+** |  |  |  |  |
| Dooley et al. (2016)/ Oral health prevention | Meso level |  |  |  |  |  |  |  | **+** | **+** | **+** |  |  |  |  |  | **+** |  |  |  | **+** |  |  |  |  |  |  |  |  |
| Yusuf et al. (2015)/ Keep Smiling | Meso level |  |  |  |  |  |  |  | **+** | **+** | **+** |  |  |  |  | **+** |  |  | **+** |  | **+** |  |  |  |  |  | **+** |  |  |

|  | | | | | | | | | | | | | | | | | | | | | | | | | | | | | |
| --- | --- | --- | --- | --- | --- | --- | --- | --- | --- | --- | --- | --- | --- | --- | --- | --- | --- | --- | --- | --- | --- | --- | --- | --- | --- | --- | --- | --- | --- |
|  |  | **Children** | | **Parents** | | | | | **Professionals** | | | **Government** | | | | **Intervention** | | | | | | | | | | | | | |
| **Authors /**  **Name**  **intervention** | **System levels(s)** | **Willingness to cooperate** | **Active involvement** | **Sharing experiences & helping others** | **Engagement and self-efficacy** | **High interest in oral health** | **Motivation to change behaviour** | **Active involvement** | **Training** | **Interdisciplinary collaboration** | **Active involvement** | **Governmental input** | **Nationwide program** | **Interdisciplinary collaboration** | **Active involvement** | **Adaptability** | **Available resources** | **Observed advantage** | **Design and content** | **Accessibility and usability** | **Feasibility** | **Population approach** | **Community approach** | **Social support / group approach** | **Personalised / family approach** | **Building rapport & trust with families** | **Educational approach for families** | **Distribution of oral health materials** | **Media use** |
| Chomitz et al. (2019)/ Baby steps to health | Meso – micro levels | \| **-** \|  \|  \|  \|  \|  \|  \|  \|  \|  \|  \|  \|  \|  \|  \|  \|  \| \| --- \| --- \| --- \| --- \| --- \| --- \| --- \| --- \| --- \| --- \| --- \| --- \| --- \| --- \| --- \| --- \| --- \| |  |  |  |  | **+** | **+** | **+** | **+** | **+** |  |  |  |  |  |  |  | **+** | **+** | **+** |  |  |  | **+** |  | **+** | **+** |  |
| Maupomé et al. (2010)/ Toddler Overweight & Tooth Decay Prevention Study | Meso – micro levels |  |  |  |  |  |  | **+** |  |  | **+** |  |  |  |  |  |  | **+** | **+** |  | **+** |  | **+** |  | **+** |  | **+** |  |  |
| Wenhall et al. (2008)/ Oral health outreach programme | Meso – micro levels | **+** |  |  |  |  |  |  |  |  | **+** |  |  |  |  |  |  | **+** | **+** | **+** |  |  |  |  | **+** |  | **+** | **+** |  |
| Neumann et al. (2011)/ Country KIDS | Meso – micro levels |  |  |  |  |  |  |  | **+** | **+** | **+** |  |  |  |  |  |  |  |  |  |  |  |  |  |  |  | **+** | **+** |  |
| Adams et al. (2017)/ Centering  Pregnancy | Meso – micro levels |  |  |  | **+** |  |  | **+** | **+** | **+** | **+** |  |  |  |  |  |  |  |  |  |  |  |  | **+** |  |  | **+** | **+** |  |

|  | | | | | | | | | | | | | | | | | | | | | | | | | | | | | |
| --- | --- | --- | --- | --- | --- | --- | --- | --- | --- | --- | --- | --- | --- | --- | --- | --- | --- | --- | --- | --- | --- | --- | --- | --- | --- | --- | --- | --- | --- |
|  |  | **Children** | | **Parents** | | | | | **Professionals** | | | **Government** | | | | **Intervention** | | | | | | | | | | | | | |
| **Authors /**  **Name**  **intervention** | **System levels(s)** | **Willingness to cooperate** | **Active involvement** | **Sharing experiences & helping others** | **Engagement and self-efficacy** | **High interest in oral health** | **Motivation to change behaviour** | **Active involvement** | **Training** | **Interdisciplinary collaboration** | **Active involvement** | **Governmental input** | **Nationwide program** | **Interdisciplinary collaboration** | **Active involvement** | **Adaptability** | **Available resources** | **Observed advantage** | **Design and content** | **Accessibility and usability** | **Feasibility** | **Population approach** | **Community approach** | **Social support / group approach** | **Personalised / family approach** | **Building rapport & trust with families** | **Educational approach for families** | **Distribution of oral health materials** | **Media use** |
| Soussou et al. (2017)/ Dental education program | Meso – micro levels |  |  |  |  |  | **+** | **+** |  |  | **+** |  |  |  |  |  |  |  | **+** |  | **+** |  |  |  | **+** |  | **+** |  |  |
| Hoeft et al. (2016)/ Contra Caries | Meso – micro levels |  |  |  |  |  |  | **+** |  |  | **+** |  |  |  |  |  |  |  | **+** |  |  |  |  | **+** |  |  | **+** |  |  |
| Huebner et al. (2014)/ Taking Care of Baby Teeth | Meso – micro levels |  |  |  | **+** |  | **+** | **+** |  |  | **+** |  |  |  |  |  | **+** |  |  | **+** |  |  |  | **+** | **+** |  | **+** | **+** |  |
| Brännemo et al. (2020)/ An extended postnatal programme | Meso – micro levels |  |  |  |  |  | **+** |  | **+** | **+** | **+** |  |  |  |  |  |  |  | **+** | **+** |  |  |  |  |  |  | **+** | **+** |  |
| Wagner et al. (2014)/ Oral health promotion programme | Meso – micro levels |  |  |  |  |  | **+** | **+** | **+** |  | **+** |  |  |  |  |  | **+** |  | **+** |  |  |  |  |  | **+** |  | **+** | **+** |  |

|  | | | | | | | | | | | | | | | | | | | | | | | | | | | | | |
| --- | --- | --- | --- | --- | --- | --- | --- | --- | --- | --- | --- | --- | --- | --- | --- | --- | --- | --- | --- | --- | --- | --- | --- | --- | --- | --- | --- | --- | --- |
|  |  | **Children** | | **Parents** | | | | | **Professionals** | | | **Government** | | | | **Intervention** | | | | | | | | | | | | | |
| **Authors /**  **Name**  **intervention** | **System levels(s)** | **Willingness to cooperate** | **Active involvement** | **Sharing experiences & helping others** | **Engagement and self-efficacy** | **High interest in oral health** | **Motivation to change behaviour** | **Active involvement** | **Training** | **Interdisciplinary collaboration** | **Active involvement** | **Governmental input** | **Nationwide program** | **Interdisciplinary collaboration** | **Active involvement** | **Adaptability** | **Available resources** | **Observed advantage** | **Design and content** | **Accessibility and usability** | **Feasibility** | **Population approach** | **Community approach** | **Social support / group approach** | **Personalised / family approach** | **Building rapport & trust with families** | **Educational approach for families** | **Distribution of oral health materials** | **Media use** |
| Hammersley et al. (2022)/ Baby teeth talk | Meso – micro levels |  |  |  | **+** |  |  | **+** | **+** | **+** | **+** |  |  |  |  | **+** | **+** | **+** | **+** | **+** |  |  |  |  | **+** | **+** |  | **+** |  |
| Gagnon et al. (2007)/ Compliance with fluoride supplements | Meso – micro levels |  |  |  |  | **+** |  | **+** |  |  | **+** |  |  |  |  |  | **+** |  |  | **+** |  |  |  |  | **+** | **+** |  |  |  |
| Purkis et al. (2023)/ Pediatric dental residency program | Meso – micro levels |  | **+** |  |  |  |  | **+** | **+** | **+** | **+** |  |  |  |  |  | **+** |  | **+** |  |  |  |  |  | **+** |  |  |  |  |
| Cunha-Cruz et al. (2017)/ Everybody Brush! | Meso – micro levels |  | **+** |  |  |  |  | **+** | **+** | **+** |  |  |  |  |  |  | **+** |  | **+** |  |  |  |  |  |  |  | **+** | **+** |  |
| Lumsden (2019) / MySmile-Buddy | Meso – micro levels |  |  |  |  | **+** |  | **+** |  |  | **+** |  |  |  |  |  |  |  | **+** |  | **+** |  |  |  | **+** |  |  |  |  |

|  | | | | | | | | | | | | | | | | | | | | | | | | | | | | | |
| --- | --- | --- | --- | --- | --- | --- | --- | --- | --- | --- | --- | --- | --- | --- | --- | --- | --- | --- | --- | --- | --- | --- | --- | --- | --- | --- | --- | --- | --- |
|  |  | **Children** | | **Parents** | | | | | **Professionals** | | | **Government** | | | | **Intervention** | | | | | | | | | | | | | |
| **Authors /**  **Name**  **intervention** | **System levels(s)** | **Willingness to cooperate** | **Active involvement** | **Sharing experiences & helping others** | **Engagement and self-efficacy** | **High interest in oral health** | **Motivation to change behaviour** | **Active involvement** | **Training** | **Interdisciplinary collaboration** | **Active involvement** | **Governmental input** | **Nationwide program** | **Interdisciplinary collaboration** | **Active involvement** | **Adaptability** | **Available resources** | **Observed advantage** | **Design and content** | **Accessibility and usability** | **Feasibility** | **Population approach** | **Community approach** | **Social support / group approach** | **Personalised / family approach** | **Building rapport & trust with families** | **Educational approach for families** | **Distribution of oral health materials** | **Media use** |
| O'Malley et al. (2017)/  Kitten’s First Tooth | Micro level | \| **-** \|  \|  \|  \|  \|  \|  \|  \|  \|  \|  \|  \|  \|  \|  \|  \|  \| \| --- \| --- \| --- \| --- \| --- \| --- \| --- \| --- \| --- \| --- \| --- \| --- \| --- \| --- \| --- \| --- \| --- \| |  |  |  |  |  |  |  |  |  |  |  |  |  |  |  |  | **+** |  |  |  |  |  | **+** |  |  |  |  |
| Lee et al (2023)/ My first teeth | Micro level |  |  | **+** |  |  | **+** | **+** |  |  |  |  |  |  |  |  |  | **+** | **+** |  |  |  |  |  | **+** |  | **+** |  | **+** |
| Al-Jallad (2022) / AICaries | Micro level |  |  |  | **+** |  |  |  |  |  |  |  |  |  |  |  | **+** |  | **+** | **+** | **+** |  |  |  |  |  | **+** |  |  |
